# Tim-3 expression on peripheral T cell subsets correlates with disease progression in hepatitis B infection

**DOI:** 10.1186/1743-422X-8-113

**Published:** 2011-03-11

**Authors:** Wei Wu, Yu Shi, Jie Li, Feng Chen, Zhi Chen, Min Zheng

**Affiliations:** 1 State Key Laboratory for Diagnosis and Treatment of Infectious Diseases, First Affiliated Hospital, School of Medicine, Zhejiang University, Hangzhou 310006, China

## Abstract

**Background and objective:**

T-cell immunoglobulin domain and mucin domain-containing molecule-3 (Tim-3) represents a novel mechanism of T-cell dysfunction in chronic viral diseases. However, the role of Tim-3 in the pathogenesis of chronic hepatitis B (CHB) is not well understood. We investigated Tim-3 expression on peripheral T cell subsets and analyzed the relationship between Tim-3 expression and disease progression in HBV infection.

**Methods:**

peripheral blood samples were obtained from CHB patients (n = 40), including 23 patients with moderate CHB [MCHB] and 17 with severe CHB [SCHB]. Control samples were obtained from nine acute hepatitis B patients (AHB) and 26 age-matched healthy subjects. The expression of Tim-3 on T cells was determined by flow cytometry.

**Results:**

Tim-3 expression was elevated on peripheral CD4^+ ^and CD8^+ ^T cells from AHB and CHB patients compared to those from healthy controls. The percentage of Tim-3^+ ^T cells was further increased in SCHB patients relative to MCHB patients and showed a positive correlation with conventional markers for liver injury (alanine aminotransferase (ALT), aspartate transaminase (AST), total bilirubin (TB) and international normalized ratio (INR) level). The frequency of Tim-3-expressing T cells was negatively correlated with T-bet mRNA expression and plasma interferon-gamma (INF-gamma) levels. Further, Tim-3 expression on CD4^+ ^or CD8^+ ^T cells was reduced in CHB patients with disease remission after antiviral treatment and in AHB patients during the convalescence phase.

**Conclusions:**

Our results suggest that over-expression of Tim-3 is involved in disease progression of CHB and that Tim-3 may participate in skewing of Th1/Tc1 response, which contributes to persistency of HBV infection.

## Introduction

Chronic hepatitis B virus (HBV) infection is a global health burden with 350-400 million people infected worldwide. Approximately 1 million deaths occur annually due to the long-term complications of infection including cirrhosis, liver failure and hepatocellular carcinoma [1]. Adaptive T-cell immunity plays a pivotal role in viral clearance [2]. Patients chronically infected with HBV present impaired T helper 1 cells (Th1) response and consequently impaired cytotoxic T lymphocyte (Tc1) priming, which results in viral persistence and liver damage [3]. In contrast those who recovered from acute HBV infection (AHB) exhibit normal Th1 and Tc1 function. The cause(s) of T-cell dysfunction in chronically infected patients have not been fully clarified.

Recent studies have focused on the role of membrane inhibitory receptors in modulating T-cell dysfunction during chronic viral diseases. Programmed cell death-1 (PD-1), a member of the CD28 family, was found to be up-regulated on the surfaces of exhausted T cells during chronic lymphocytic choriomeningitis virus (LCMV) [4], human immunodeficiency virus (HIV) [5], hepatitis C virus (HCV) [6] and HBV infections [7]. Furthermore, blockade of this pathway *ex vivo *or *in vivo *significantly improved T-cell function. However, T-cell exhaustion was not always completely corrected by blocking the PD-1 pathway, suggesting that other pathways may involved in T-cell dysfunction [8].

T-cell immunoglobulin domain and mucin domain-containing molecule-3 (Tim-3) was first found to be expressed on Th1 but not Th2 cells [9]. Tim-3 negatively regulates Th1 response and induces tolerance through the Tim-3/Galectin-9 pathway in autoimmune diseases [10]. Tim-3 is highly expressed on effector T cells during HIV and HCV infection, and blocking the Tim-3 signaling pathway restores proliferation and enhanced cytokine production of effector T cells [11,12]. Furthermore, a synergistic effect was observed by blocking both the Tim-3 and PD-1 pathway during chronic LCMV infection [13]. Taken together, these results indicated that Tim-3 might be another negative regulatory receptor contributing to T-cell dysfunction.

However, the role of Tim-3 in HBV infection has not been clarified. Therefore we investigated the expression of Tim-3 on peripheral T cells in patients chronically infected with hepatitis B and tested whether the expression level of Tim-3 correlates with disease progression.

## Materials and methods

### Subjects

Samples were obtained from HBV-infected patients admitted to the infectious department of our hospital, including with 23 with mild CHB (MCHB), 17 with severe CHB (SCHB), 9 with AHB and 26 healthy controls. The demographic features of study group are presented in Table 1. The mean age of SCHB patients was higher than that of the other three groups. There was no significant difference in sex proportion among the groups.

**Table 1:** Clinical characteristics of the studied groups

Variables	N.C. (N = 26)	AHB (N = 9)	MCHB (N = 23)	SCHB (N = 17)
Age (year)	30.2 ± 5.4	32.7 ± 13.5	33.1 ± 11.4	42.8 ± 13.1
Sex	69.2	88.9	70.0	70.6
HBsAg (%)	0	77.8	100	100
HBsAb (%)	61.5	22.2	0	0
HBeAg (%)	0	77.8	50	35.7
HBeAb (%)	3.8	22.2	50	71.4
HBcAb (%)	34.6	100	100	100
Log HBV DNA	NA	4.66 ± 2.95	6.87 ± 1.59	4.34 ± 3.07
ALT (IU/L)	15.3 ± 9.2	1197.5 ± 804.7	360.6 ± 344.6	487.8 ± 775.9
AST (IU/L)	11.7 ± 6.8	537.1 ± 539.0	241.7 ± 261.3	417.2 ± 597.1
Bilirubin (umol/L)	14 ± 11.1	113.2 ± 105.6	65.6 ± 55.2	332.5 ± 179.6
INR	NA	1.14 ± 0.26	1.02 ± 0.12	2.32 ± 0.85

Data were expressed as mean ± SD or percentage; N.C.: normal control; AHB: acute hepatitis B; MCHB: moderate chronic hepatitis B; SCHB: severe chronic hepatitis B; ALT: alanine aminotransferase; AST: aspartate transaminase; INR: international normalized ratio.

The criteria for diagnosis of AHB and CHB were described in detail previously [14]. SCHB was diagnosed if serum total bilirubin (TBIL) [15] exceed 10 times of normal upper limit (171 uM) and prothrombin time activity (PTA) exceed 40%, while those with TBIL less than 171 uM and PTA less than 40% were diagnosed as MCHB. We excluded individuals co-infected with hepatitis C virus (HCV), hepatitis D virus (HDV) and human immunodeficiency virus (HIV); persons with other causes of chronic liver diseases such as autoimmune hepatitis and Wilson disease; patients with suspected signs of hepatocellular carcinoma (HCC) by ultrasound or serum AFP; and anyone who received antiviral treatment within the previous 6 months. This study was approved by the local ethics committee and we obtained written informed consent from each study participant. The study was carried out in accordance with Declaration of Helsinki.

### Virological assessment

Serological markers of HBV, HCV, HDV and HIV were tested by commercial enzyme immunoassay kits (AXSYM System, Abbott, Wiesbaden, Germany) as previously described [16]. Serum HBV DNA level was determined using a quantitative polymerase chain reaction (qPCR) assay (PG Biotech, Shenzhen, China). The lower detection limit threshold is 500 copies/ml.

### PBMC isolation

Peripheral blood mononuclear cells (PBMCs) were isolated from fresh-heparinized blood by standard Ficoll-Hypaque density centrifugation (Biochrom, Berline, Germany). PBMC isolation was performed no more than 2 hours after peripheral blood was collected by venipuncture.

### Flow cytometric analysis

For Tim-3 expression analysis, freshly-isolated PBMCs were stained with anti-CD3-APC, anti-CD4-FITC or anti-CD8-FITC (BD Biosciences) and anti-TIM-3-PE (R&D Systems). To exclude nonspecific binding, isotype-matched antibodies (Beckman Coulter) were used as controls. At least 1×10^5 ^cells were analyzed using a Beckman Coulter flow cytometer (FC500 MPL, Fullerton, CA, USA).

### Quantification of T-bet mRNA by real-time PCR

Total RNA was extracted from freshly isolated PBMCs according to the manufacturer's protocol (Invitrogen, Carlsbad, CA). The T-bet mRNA was determined by performing a real-time PCR with SYBR-green I Premix ExTaq on the ABI Prism 7900 (Applied Bio systems, Foster, CA). The primer pairs were used as follow: T-bet-forward: TGTGACCCAGATGATTGTGC; T-bet-reverse: AAAGATATGCGTGTTGGAAGC; GAPDH-forward: GGTGGTCTCCTCTGACTTCAACA; GAPDH-reverse: GTTGCTGTAGCCAAATTCGTTGT. All PCR assays were performed in duplicate, and data were analyzed with the ABI Prism Detection system using the comparative threshold cycle method as previously described. GAPDH was used as internal control and RNA samples from the healthy groups were used as quality control in each RT-PCR.

### Quantification of plasma INF-gamma by flow-cytomix

Plasma INF-gamma level was determined using the flow-cytomix simplex kit, according to the manufacturer's instructions (Bender MedSystem, Copenhagen, Denmark). The detection thresholds were 1.6 pg/ml.

### Statistical analysis

Data were expressed as mean ± SD or number (%). The Mann-Whitney U test and Chi-Square test were used to compare differences among the study groups. The paired t test was used to compare differences in Tim-3 expression before and after treatment, at early phase, and at convalescence stage. Spearman correlation was conducted to assess the association between the frequency of Tim-3-expressing T cells and the other indicated parameters. A P value of less than 0.05 was considered to be statistically significant. All statistical analysis was performed with SPSS 16.0 for Windows (SPSS, Chicago, IL).

## 2 Results

### 2.1 Up-regulation of Tim-3 expression on peripheral T-cell subsets in HBV-infected subjects

**Figure 1:**
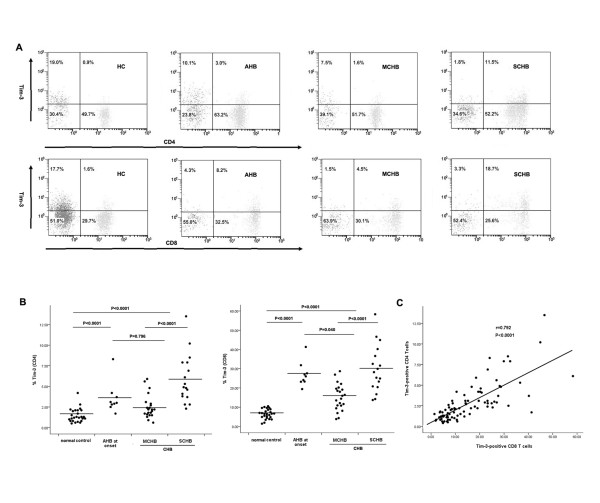
**Elevated Tim-3 expression on circulating CD4 and CD8 T cells in HBV-infected individuals**. (A) PBMCs from normal controls, AHB patients (early phase), MCHB and SCHB patients were stained with antibodies against CD3, CD4/CD8, and Tim-3. An isotype-matched antibody was used as a negative control. (B) The percentage of Tim-3 cells within CD4+ and CD8+ T cell populations are increased in AHB and CHB patients and further enhanced in the severe stage of CHB. Each dot represents an individual data point and the horizontal lines represent the mean. The Mann-Whitney U test was used to compare differences among groups. (C) There is a significantly positive correlation between the frequency of Tim-3-expressing CD4^+ ^and CD8^+ ^T cells in the studied subjects. Spearman test was performed for correlation analysis.

We examined Tim-3 expression from patients in the three groups by flow cytometry on PBMCs using an anti-Tim-3 polyclonal antibody. An elevated frequency of Tim-3-positive CD4^+ ^cells was observed in subjects with AHB (onset) and CHB compared to non-infected controls (1.48 ± 0.83% for normal controls versus 3.58 ± 1.90% for patients with AHB [P < 0.0001], 3.93 ± 2.76% for CHB patients [P < 0.0001], Figure 1A and 1B). We also observed increased Tim-3 expression on CD8^+ ^T cells from AHB and CHB patients, relative to normal controls (6.43 ± 2.50% for controls versus 27.12 ± 6.33% for AHB patients [P < 0.0001] and 21.34 ± 11.95% for CHB patients, Figure 1A and 1B). Patients with AHB presented higher Tim-3 expression on CD8^+ ^(P = 0.040) but not CD4^+ ^T cells (P = 0.796) relative to CHB patients. Tim-3 expression on both CD4^+ ^(P < 0.0001) and CD8^+ ^(P < 0.0001) T cells was significantly elevated in SCHB patients compared to that in MCHB patients. Furthermore, the frequency of Tim-3-expressing CD4^+ ^T cells correlated significantly with Tim-3 expression on CD8^+ ^T cells (r = 0.792, P < 0.0001, Figure 1C). Taken together these results indicate that Tim-3 expression is increased in parallel with disease severity.

### 2.2 The frequency of Tim-3-expressing T cells correlates with hepatic injury but not viral load in CHB patients

**Figure 2:**
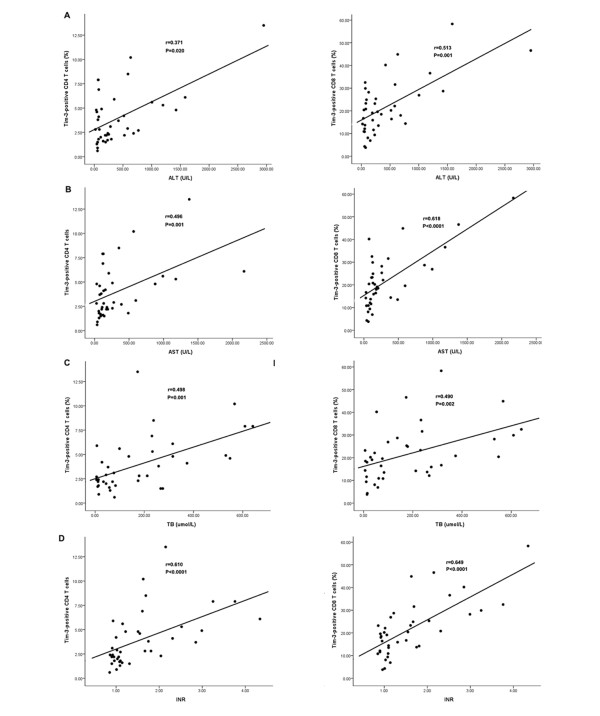
**Association between the frequency of Tim-3^+ ^T cells and conventional markers for liver damage in CHB patients**. The frequency of CD4^+ ^or CD8^+ ^T cells expressing Tim-3 are positively correlated with the levels of laboratory parameters for assessing liver injury such as ALT (A), AST (B), TB (C) and INR (D). Spearman test was used for correlation analysis.

We then evaluated the correlation between Tim-3 expression and established markers for liver damage. We found that the frequency of Tim-3^+ ^CD4^+ ^or CD8^+ ^T cells significantly correlated with levels of alanine aminotransferase (ALT) (r_CD4 _= 0.371, P_CD4 _= 0.020; r_CD8 _= 0.371, P_CD8 _= 0.001), aspartate transaminase (AST) (r_CD4 _= 0.496, P_CD4 _= 0.001; r_CD8 _= 0.618, P_CD8 _< 0.0001), international normalized ratio (INR) (r_CD4 _= 0.498, P_CD4 _= 0.001; r_CD8 _= 0.490, P_CD8 _= 0.002), and total bilirubin (r_CD4 _= 0.610, P_CD4 _< 0.0001; r_CD8 _= 0.649, P_CD8 _< 0.0001) (Figure 2). These results suggest that Tim-3 could be used as a potential marker for assessing severity of hepatic injury in CHB. As it was reported that Tim-3 expression was associated viral load during HIV infection, we examined whether this relationship was established in chronic HBV infection. However, we did not observe a significant correlation between serum HBV DNA with the frequency of either Tim-3+ CD4^+ ^T cells (r_CD4 _= -0.211, P_CD4 _= 0.204) or Tim-3+ CD8^+ ^T cells (r_CD4 _= -0.206, P_CD4 _= 0.215).

### 2.3 The frequency of Tim-3-positive T cells negatively correlates with T-bet mRNA expression in CHB patients

**Figure 3:**
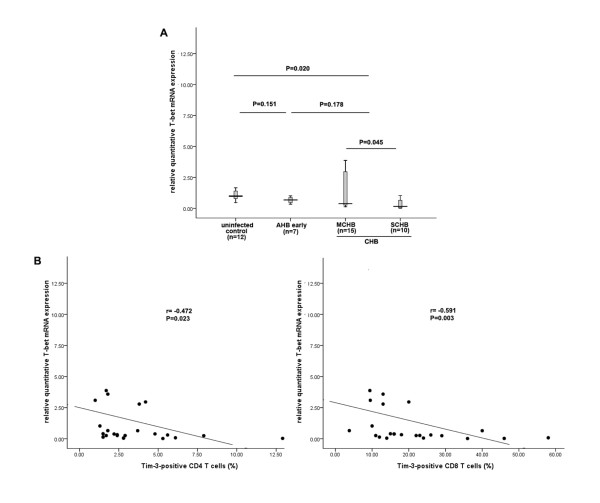
**Association between the frequency of Tim-3^+ ^T cells and T-bet mRNA expression in CHB patients**. (A) T-bet mRNA expression was determined in patients separated into four groups: normal controls (N = 12), AHB patients (early phase) (N = 7), MCHB patients (N = 15) and SCHB patients (N = 10). The bars represent the (95% CI) T-bet mRNA expression for each group and the differences among groups were analyzed using the Mann-Whitney U test. (B) Spearman correlation analysis showed a negative association between the frequency of Tim-3-postivie T cells and T-bet mRNA expression.

Tim-3 was reported to negatively regulate the Th1/Tc1 response. As T-bet was a Th1/Tc1-related transcription factor, we determined T-bet mRNA expression in PBMCs from healthy controls, AHB patients and CHB patients. The relative amount of T-bet mRNA expression in patients was 0.89 fold of healthy controls (P = 0.020). However, there was no significant difference between CHB (0.89 fold of healthy controls) and AHB patients (0.86 fold of healthy controls) (P = 0.178). Lower T-bet mRNA expression was observed in SCHB patients (0.53 fold of healthy controls) compared to MCHB patients (1.11 fold of health controls) (P = 0.045) (see Figure 3A). Moreover, we observed a significant inverse correlation between T-bet mRNA expression and the frequency of Tim-3^+ ^T cells (r_CD4 _= -472, P_CD4 _= 0.023; r_CD8 _= -0.591, P_CD8 _= 0.003) (see Figure 3B). However, these correlations were not observed in AHB patients (data not shown).

### 2.4 The frequency of Tim-3-positive T cells negatively correlates with plasma IFN-gamma level in CHB patients

**Figure 4:**
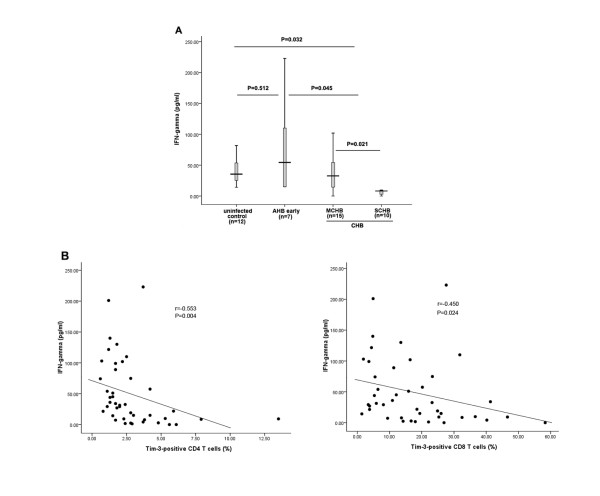
**Association between the frequency of Tim-3^+ ^T cells and plasma IFN-gamma level in CHB patients**. (A) IFN-gamma was measured in plasma from normal controls (N = 12), AHB patients (early phase) (N = 7), MCHB patients (N = 15) and SCHB patients (N = 10). The bars represent the (95% CI) IFN-gamma level for each group and the differences among groups were analyzed using the Mann-Whitney U test. (B) Spearman correlation analysis showed there were negative associations between the frequency of Tim-3-postivie T cells and plasma IFN-gamma level.

We next assessed plasma levels of IFN-gamma, a Th1/Tc1-related cytokine, in 12 uninfected controls, 7 AHB patients and 25 CHB patients. Plasma IFN-gamma concentration were significantly lower in CHB patients relative to AHB patients and controls (INF-gamma: 28.16 ± 34.51 pg/ml for CHB patients versus 78.63 ± 79.92 pg/ml for AHB patients [P = 0.045], 40.82 ± 21.58 for normal controls [P = 0.032]) (see Figure 4A). Moreover, we observed a significant inverse correlation between plasma IFN-gamma and the frequency of Tim-3^+ ^T cells (r_CD4 _= -0.553, P_CD4 _= 0.004; r_CD8 _= -0.450, P_CD8 _= 0.024) (see Figure 4B) Again, these correlations were not observed in AHB patients (data not shown).

### 2.5 Tim-3 expression was reduced in CHB patients with disease remission

**Figure 5:**
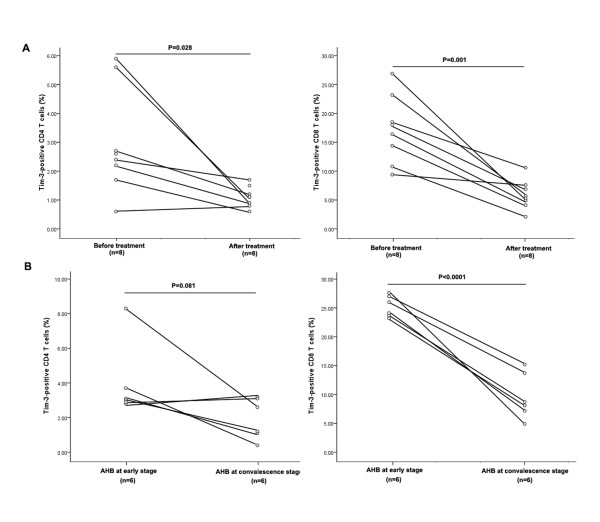
**longitudinal analysis of Tim-3 expression in CHB and AHB patients**. (A) The percentage of Tim-3^+ ^T cells decreased in CHB patients after successful antiviral treatment. The frequency of Tim-3^+ ^cells in CD4^+ ^and CD8^+ ^T cells before and after treatment is shown. (B) The frequency of Tim-3^+ ^T cells decreased in AHB patients during disease convalescence. The frequency of Tim-3^+ ^cells in CD4^+ ^and CD8^+ ^T cells at early stage and convalescence stage is shown.

The previous results indicated a role of Tim-3 in disease progression of CHB. To further evaluate this hypothesis, we conducted a longitudinal study on Tim-3 expression on T cells from 8 CHB patients before and after antiviral treatment with a 3-6 month follow-up time. All the 8 patients achieved complete biochemical (ALT normalization) and virological response (suppression of serum HBV DNA to undetectable levels) after 6-months of antiviral treatment. As shown in Figure 5A, Tim-3 expression on both CD4^+ ^and CD8^+ ^T cells was significantly reduced in these patients (CD4: 2.96 ± 1.85% for early phase versus 1.11 ± 0.36% for convalescence phase, P = 0.028; CD8: (17.20 ± 5.89% for early phase versus 5.90 ± 2.54% for convalescence phase, P = 0.001)).

### 2.6 Tim-3 expression was reduced in AHB patients at convalescence stage

We also performed follow-up of 6 AHB patients to analyze the dynamic alteration of Tim-3 expression with disease convalescence. AHB convalescence was defined as ALT normalization and HBsAg-seroconversion. As shown in Figure 5B, the Tim-3^+ ^CD8^+ ^T cell frequency of the AHB patients was high in the early acute phase and significantly reduced in the convalescent phase (25.27 ± 1.85% for early phase versus 3.98 ± 1.62% for convalescence phase, P < 0.0001). Tim-3 expression on CD4^+ ^T cells also appeared to be reduced although this effect did not quite achieve statistical significance (3.97 ± 2.14% for early phase versus 1.93 ± 1.18% for convalescence phase, P = 0.081).

## Discussion

Tim-3, expressed in both innate and adaptive immune cells, plays a pivotal role in immune regulation and immune tolerance [17]. A recent study showed that HBV infection can up-regulate Tim-3 expression in NK cells, which may in turn suppress NK-cell functions in CHB patients [18]. Viral clearance and liver damage is thought to be mediated primarily through T cell-mediated responses [19], however; up to now, no study investigated the role of Tim-3 in regulating T-cell immunity in CHB patients. In this study, we determined the expression profile of Tim-3 on peripheral T cell subsets from CHB and AHB patients and investigated the potential role of Tim-3 in disease progression.

We first demonstrated increased Tim-3 expression on CD4^+ ^and CD8^+ ^T cells in patients with CHB and found that Tim-3 expression was further enhanced in samples from patients with more severe stages of CHB. Moreover, there was a significant positive correlation between Tim-3 expression with conventional markers for hepatic injury such as ALT, AST, INR and TB. These results indicate that Tim-3 expression level is related to the degree of liver inflammation. This was further supported by an observed decrease of Tim-3 expression in CHB patients with disease resolution. Since activated T cells cause liver injury, and the severity of liver inflammation is correlated with the number of intra-hepatic activated T cells [19], up-regulation of Tim-3 expression might be due to the activation of T cells.

Accumulating evidence indicates that Tim-3 could negatively regulate adaptive immune response. Upon interaction with its ligand, Galectin-9, Tim-3 induces Th1 cell death and suppresses IFN-gamma production [10]. Further, IFN-γ secretion from CD4^+ ^and CD8^+ ^T cells could be restored by blockade of the Tim-3/Galectin-9 pathway or by Tim-3 knockdown using specific shRNAs [11]. In this study, we demonstrated lower expression of T-bet mRNA and lower levels of plasma IFN-gamma in CHB patients compared with AHB patients or uninfected controls, suggesting an impaired Th1/Tc1 response in CHB patients. We further found an inverse correlation between Tim-3 expression and both T-bet expression and plasma IFN-gamma, in CHB patients but not AHB patients. These results provided suggestive evidence that Tim-3 down-regulates the Th1/Tc1 response, which might contribute to viral persistence during chronic HBV infection.

Previous work showed that Tim-3 was highly expressed on CD4^+ ^and CD8^+ ^T cells in acutely/early HIV infected individuals^11^. Similarly, we also observed high expression of Tim-3 on T cells during the early stage of acute HBV infection. However, in AHB patients, Tim-3 was expressed as a transient up-regulation, which subsequently decreased at convalescence stage. This Tim-3 expression pattern was in line with that of PD-1 observed during acute-limited HBV infection [20]. Tim-3 expression was not associated with severity of hepatic injury in AHB patients. Furthermore, unlike CHB, there was no association between Tim-3 expression and IFN-gamma or TNF-alpha level in AHB patients. Overall, these results suggested a different role of Tim-3 in the pathogenesis of AHB and CHB, which might determine the diversification of HBV infection.

## Conclusions

Collectively, the expression of Tim-3 is up-regulated on circulating CD4^+ ^and CD8^+ ^T cells in CHB patients. Tim-3 was highly expressed on T cells from AHB patients as well, however, its expression decreased dynamically in convalescence phase. Tim-3 expression positively correlated with disease severity and negatively correlated with Th1/Tc1 response in CHB patients. Our data suggest that Tim-3 might be a potential marker for evaluating severity of hepatic flare-up. However, further studies were needed to clarify the exact role of Tim-3 in the pathogenesis of CHB.

## List of abbreviations

(Tim-3): T-cell immunoglobulin domain and mucin domain-containing molecule-3; (CHB): chronic hepatitis B; (MCHB): moderate chronic hepatitis B; (SCHB): severe chronic hepatitis B; (AHB): acute hepatitis B; (ALT): alanine aminotransferase; (AST): aspartate transaminase; (TB): total bilirubin; (INR): international normalized ratio; (INF-gamma): interferon-gamma.

## Competing interests

No benefits in any form have been received or will be received from a commercial party related directly or indirectly to the subject of this article.

## Authors' contributions

WW, SY, CF, CZ and ZM designed the study. WW, SY and LJ participated in conducting experiments. WW, SY, CF and ZM performed statistical analysis and data interpretation. WW, SY and LJ wrote the main body of the article under the supervision CF, CZ and. ZM. All authors have read and approved the final manuscript.
